# Semisynthetic aurones A14 protects against T-cell acute lymphoblastic leukemia via suppressing proliferation and inducing cell cycle arrest with apoptosis

**DOI:** 10.1186/s13020-022-00693-6

**Published:** 2022-12-12

**Authors:** Meng Wang, Lisi Li, Tengyun Fan, Lixue Cao, Jiayi Zhang, Shuang Li, Chunming Liu, Xifu Liu

**Affiliations:** 1 Ministry of Education Key Laboratory of Molecular and Cellular Biology, Hebei Anti-Tumor Molecular Target Technology Innovation Center, College of Life Science, Hebei Normal University, Shijiazhuang, China; 2 Department of Molecular and Cellular Biochemistry, College of Medicine, University of Kentucky, Lexington, KY USA; 3 Jianyuan Science & Technology (Zhangjiakou) Co., Ltd., Zhangjiakou, China

**Keywords:** Semisynthetic aurones A14, T-cell acute lymphoblastic leukemia, Cell proliferation, Cell cycle, Apoptosis

## Abstract

**Background:**

Acute lymphoblastic leukemia is an aggressive neoplasm and seriously threatens human health. A14 is one kind of semisynthetic aurone that exhibits the capability to inhibit prostate cancer, but little is known about the role of A14 on T-cell acute lymphoblastic leukemia.

**Methods:**

Firstly, the effects of A14 on the ability of leukemia cells to proliferate were measured by Vi-cell counter. Then, we detected the cell cycle and apoptosis by flow cytometry and characterized the related protein expression using immunoblotting. In addition, we constructed stable luciferase expressing cell lines for use in a cell derived xenograft mouse model to measure the effect of A14 on T-cell acute lymphoblastic leukemia.

**Results:**

Results exhibited that A14 markedly suppressed cell proliferation and induced G2/M phase arrest along with cell cycles regulating proteins changes. A14 led to apoptosis in leukemia cells, at least partly, through the cytochrome c signaling pathway. Experiments in cell derived xenograft mouse model also showed that A14 markedly ameliorated the survival rate.

**Conclusions:**

The present study revealed that semisynthetic aurones A14 can effectively protect against T-cell acute lymphoblastic leukemia progression both in vitro and in vivo, indicating the capability of A14 as a promising drug for the treatment of T-cell acute lymphoblastic leukemia.

## Background

As a hematological disease, acute lymphoblastic leukemia (ALL) is a heterogeneous malignancy. ALL is one of the most common forms of leukemia and is mainly induced from hematopoietic precursors of lymphoid cells including B-cell acute lymphoblastic leukemia (B-ALL) or T-cell acute lymphoblastic leukemia (T-ALL). T-ALL is a lymphoblasts' neoplasm, whose genesis is associated with the lesion of several tissues including blood, bone marrow, lymph nodes, thymus and so on [1]. As an aggressive neoplasm, T-ALL affects both children and adults [2]. T-ALL accounts for approximately 10–15% of pediatric patients and nearly 25% of adult patients in ALL cases. Due to its unique clinical features, T-ALL has attracted more and more attention in recent years. Patients with T-ALL have high rates of relapse, while also being poor responders to chemotherapy drugs [3]. Compared with B-ALL, T-ALL demonstrates a less favorable prognosis. Following improvements in T-ALL therapy, the event-free survival for 5 years in T-ALL patients has increased, but the survival rate is still only about 30–40% in adult patients and 60–70% in pediatric patients [4, 5]. As it stands, chemotherapeutic regimens remain critical for the treatment of T-ALL patients, however, the short-term and long-term side effects of chemotherapeutic treatment hasten the discovery of new drugs or advanced treatments to solve the clinical challenge.

A common characteristic of cancer cells is the mitotic catastrophe, which is usually associated with DNA damage [6]. The formation of mitotic spindle and overexpression of cell cycle proteins promote cancer cell mitosis, leading to cell proliferation. It has been widely accepted that chemotherapeutic drugs inducing cell cycle arrest can be used as potential drugs for the treatment of cancer. Cell cycle contains the interphase and mitotic phase, meanwhile, the interphase can be divided into the G1 phase, S phase and G2 phase. The cell cycle checkpoints play a pivotal role in DNA repair, and the cell cycle phase transitions are regulated by mitosis-promoting factors including cyclins and cyclin-dependent kinases (CDKs) [7]. Aside from cyclins and CDKs, p53 has also been demonstrated to regulate the G2 checkpoint and activate p21 [8, 9].

In tumor development, cell apoptosis also plays an important role. Several proteins can induce cell apoptosis through proteolysis of specific targets, such as poly ADP-ribose polymerase (PARP). Among these proteins, cysteine proteases, which are members of the caspases family, can be activated by proapoptotic signal and induce an apoptosis signal cascade in cancer cells [10]. Because the caspase family can be divided into initiator caspases and effector caspases, there are several different apoptosis signals in the caspases family. Caspase-8, caspase-9 and caspase-12 belong to the initiator caspases, while caspase-3, caspase-6 and caspase-7 belong to the effector caspases. The initiator caspases are activated by the apoptotic stimuli, which in turn act on the effector caspases and promote the process of cell death [11, 12].

The uncommon flavonoid class of aurones has garnered increasing attention in recent years [13]. As a secondary metabolite from natural products, aurones are classified into the flavonoids family. The flavonoids family also contains flavones, flavanones, catechins and others [14, 15]. It has been found that aurones are widely present in vegetables, fruits and flowers, and contribute to the pigmentation of plants [16]. Notably, the privileged structure of aurones allow for a wide range of biological activities, such as antioxidant, antibacterial, antiinflammatory and so on [16, 17]. (Z)-2-((2-((1-Ethyl-5-methoxy-1H-indol-3-yl)methylene)-3-oxo-2,3-dihydrobenzofuran-6-yl)oxy) acetonitrile, also known as A14, is one kind of semisynthetic aurones. Previous studies have reported the inhibition effect of A14 on PC-3 tumor xenografts in nude mice [18], but little is known about the role of A14 on T-cell acute lymphoblastic leukemia mice. In this study, the effects of A14 on T-cell acute lymphoblastic leukemia were elevated in vitro and in vivo.

## Materials and methods

### Compounds

To synthesize (Z)-2-((2-((1-Ethyl-5-methoxy-1H-indol-3-yl)methylene)-3-oxo-2,3-di-hydrobenzofuran-6-yl)oxy) acetonitrile (A14), 6 mmol anhydrous potassium carbonate was added to 2 mmol of (2Z)-2- [(1-ethyl-5-methoxy-1 h-indole-3-yl)meth-ylene]-6-hydroxy-1-benzofuran-3(2*H*)-one in 10 mL n,n-dimethylformamide (DMF) solution. The mixture was heated to 60 °C, mixed with 2.4 mmol/L chloroacetonitrile and stirred at 60 °C for another 8 h before being cooled. The resulting mixture was then added to 100 mL of 0.05 mol/L sulfuric acid aqueous solution. The precipitate was collected by filtration, washed with water, dried and recrystallized in DMF methanol to obtain A14 yellow crystal: mp 230–232 °C.

A14 was dissolved in dimethyl sulfoxide (DMSO, Sigma-Aldrich, St. Louis, MO, USA) and prepared into corresponding concentrations for cell experiments. In the animal experiments, A14 was dissolved in solvent (50% PEG-400 + 35% PBS + 10% DMSO + 5% Tween-80) to prepare a 0.2% A14 solution. Briefly, A14 was added to 10% DMSO, mixed well, added to 50% PEG-400 and 5% Tween-80, mixed ultrasonically for 2 h, supplemented with 35% PBS, and stirred using a vortex mixer before use.

### Cell culture

Human leukemia cell lines Jurkat and THP-1, purchased from China Fenghui Biotechnology Co., Ltd. Cells were cultured in RPMI-1640 and supplemented with 10% fetal bovine serum (FBS), 100 mg/mL streptomycin and 100 U/mL penicillin (Gibco Life Technologies, Carlsbad, CA, USA). The cells were kept in a humid incubator at 37 °C with 5% CO_2_.

### Cell proliferation assay

We detected the proliferation of Jurkat and THP-1 cells according to the protocol. Cells were treated with different concentrations of A14 (10 μM, 3 μM, 1 μM, 0.3 μM, 0.1 μM, 0.03 μM, 0.01 μM), and each concentration was repeated three times. Equal volumes of DMSO were added to the blank wells. After 5 days of treatment, Vi-cell (Beckman Coulter, Indianapolis, IN, USA) counter was used to calculate the inhibition rate.

### Colony formation assay

1.2% and 0.7% agar were prepared and maintained at 42 °C without solidification after high-pressure sterilization.1.2% agar mixed with RPMI 1640 medium (RPMI 1640 + 20% FBS + 2% PS), was laid on a 6-well plate as the lower glue and incubated at 37 °C for 30 min to fully solidify. The prepared Jurkat or THP-1 cell suspension were mixed with 0.7% agar. The mixed medium was incubated in a 6-well plate as the upper glue. After solidification, the complete medium was supplemented with corresponding A14, and the culture medium was changed every 3 days. After 2 weeks, the clone size was stained with 0.1% crystal violet (Sigma-Aldrich) and observed by Nikon ECLIPSE Ts2 light microscope (Nikon Corporation, Tokyo, Japan).

### Cell cycle assay

We measured the cell cycle of Jurkat and THP-1 cells according to the instructions of cell cycle kit. Cells with a density of 1 × 10^6^/well were inoculated into 6-well plates, and different concentrations of A14 were added to each well for treatment. The blank control well was treated with DMSO. After 24 h of treatment, cells were collected and transferred to a centrifuge tube, supernatant was discarded, and washed with PBS. To detect the cell cycle by flow cytometry (Beckman Coulter, Indianapolis, IN, USA), DNA Staining solution and permeability solution were added to each tube, mixed well with a vortex and incubated at room temperature in darkness for 30 min.

### Cell apoptosis analysis

We also measured the apoptosis of Jurkat and THP-1 cells via the apoptosis kit. Cells with a density of 1 × 10^6^ were incubated into 6-well plates and challenged with different concentrations of A14. After 24 h of treatment, the cells were collected by centrifugation. After washing the cells with precooled PBS, we added AnnexinV-FITC and propidium iodide (PI) (BioLegend Inc., San Diego, CA, USA Biolegend, San Diego, CA) into each tube, and incubated them at room temperature for 5 min. We were then able to detect AnnexinV-FITC by flow cytometry (Beckman Coulter).

### Cell mitochondrial membrane potential analysis

As above, Jurkat cells and THP-1 cells were stimulated with corresponding concentrations of A14. Carbonyl cyanide 3-chlorophenylhy drazone (CCCP) was added to the cell culture medium and treated for 20 min as a positive control. Cells were stained with 5,5′,6,6′-Tetrachloro-1,1′,3,3′-tetraethyl-imidacarbocyanine iodide (JC-1) and incubated at 37 °C for 20 min. We then washed the cells with JC-1 staining buffer five times, and analyzed them using cell flow cytometry (Beckman Coulter).

### Cell derived xenograft model

First, we constructed a cell line with stably expressing luciferase gene. Specifically, Jurkat cells were cultured in 96 well plates with 5% virus per well. After incubation at 37 °C for 6 h, the medium was changed to 2 μg/mL puromycin for continuous screening over several days. Then the monoclonal antibodies were selected and isolated.

Female NOD/SCID mice aged 6–8 weeks were fed in a SPF room with free access to water and food. Mice were divided into five groups randomly and injected with 1 × 10^6^ Jurkat-Luc (luciferase reporter gene) cells per mouse through the tail vein. Three days later, the fluorescence was detected by IVIS Lumina Series III (PerkinElmer, Waltham, MA, USA) to judge whether the CDX model was established successfully. The mice that successfully expressed fluorescence were randomly divided into A14 (20 mg/kg) group and control group (n = 15). The weights of the mice were recorded every day, and the fluorescence was detected every five days. All the animal procedures were performed in strict conformance to the Guidelines for Care and Use of Laboratory Animals of Hebei Normal University and approved by the Animal Ethics Committee of Hebei Normal University.

### Immunoblot analysis

Total proteins were extracted from cells using RIPA buffer with cocktail and phenylmethanesulfonyl fluoride (Cell Signaling Technology, Inc, Boston, MA, USA). The protein content was determined by the BCA protein assay kit (Beyotime Institute of Biotechnology, Shanghai, China). Aliquots were separated on 10% sodium dodecyl sulphate–polyacrylamide (SDS-PAGE) gel, and then transferred to the polyvinylidene fluoride (PVDF) membrane (EMD Millipore, Billerica, MA, USA). After being sealed with 5% milk, the membrane was washed with TBST and incubated overnight with primary antibodies at 4 °C. The primary antibodies include CDK1 (1:1000, Abcam, Cambridge, MA, USA), cyclin B1 (1:1000, Abcam), CDK2(1:1000, Abcam), cyclin D1 (1:1000, Cell Signaling Technology, Inc., Danvers, MA, USA), Caspase 3 (1:1000, Cell Signaling), Cleaved Caspase 3 (1:1000, Cell Signaling), Caspase 9 (1:1000, Cell Signaling), Cleaved Caspase 9 (1:1000, Cell Signaling), poly [ADP-ribose] polymerase (PARP) (1:1000, Cell Signaling), Bcl-2 (1:1000, Abcam) and GAPDH (1:1000, Cell Signaling). The membrane was washed three times and incubated with the horseradish peroxidase (HRP)-conjugated secondary antibody (1:10,000, Abcam) at room temperature for one hour. ChemiDoc^MP^ Imaging Systems (BioRad Laboratories, Inc., Hercules, CA, USA) used an enhanced chemiluminescence detection kit (Beyotime Institute of Biotechnology) to detect the protein band. The optical density of protein bands were quantified by the Quantity One software (Bio-Rad), and the values were expressed as the ratio of protein to GAPDH with the values of the DMSO group set to one.

### Statistical analysis

Statistical analyses were performed by one-way analysis of variance (ANOVA) with Tukey’s post hoc test. All results were presented as the mean ± standard deviation (SD), *p* < 0.05 was thought to be statistically significant.

## Results

### A14 suppressed leukemia cell proliferation

**Fig. 1:**
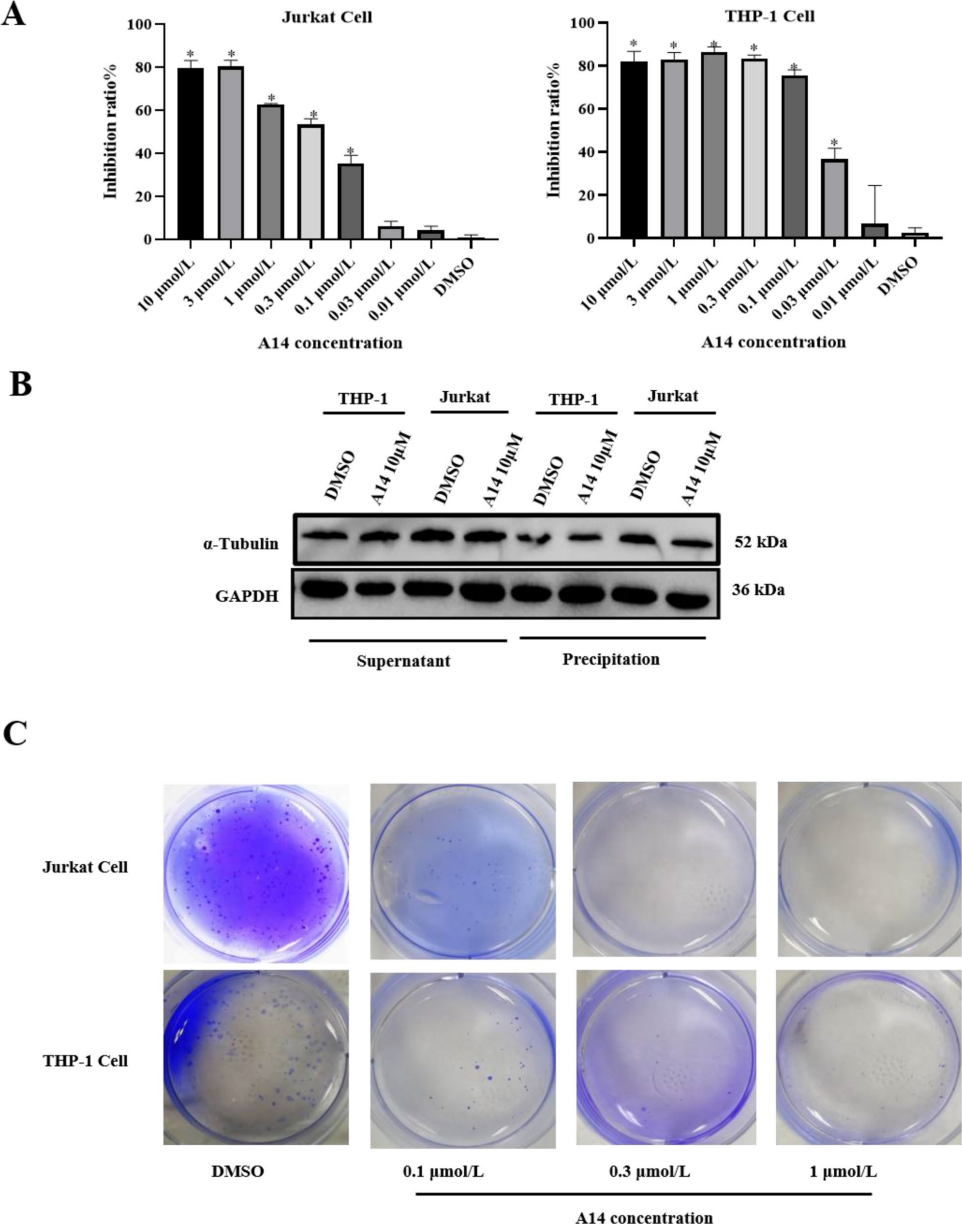
Effects of A14 on leukemia cells. **A** Inhibition effects of A14 on Jurkat cells and THP-1 cells viability. **B** Immunoblot analysis of α-tubulin in Jurkat cells and THP-1 cells. **C** The number of cell clones of Jurkat cells and THP-1 cells with A14 challenged. n = 3–4, values represent the means ± SD. *p < 0.05 *vs.* DMSO group

Firstly, we investigated the effect of A14 on the cell proliferation ability of leukemia cells. Results showed that A14 inhibited Jurkat cell proliferation by 50% at 0.3 μM and THP-1 cells by 40% at 0.03 μM concentration (Fig. 1A). As we have demonstrated that A14 is a tubulin inhibitor in PC-3 cells [18], we wonder whether the proliferation inhibition is associated with the tubulin inhibitor. Results demonstrated A14 decreased tubulin polymerization especially in Jurkat cells, indicating that tubulin inhibition may be contributed the cell proliferation by the A14 (Fig. 1B). In line, the soft agar cloning experiment showed that cell cloning in the 0.1 μM concentration is less than in the control group, and there is nearly no cell cloning formation in both 0.3 μM and 1 μM A14-treated groups, suggesting that A14 can significantly inhibit the cloning formation capacity in leukemia cells (Fig. 1C). These data suggested that A14, as a tubulin inhibitor, can effectively suppress leukemia cell proliferation.

### A14 treatment induces G2/M phase arrest

**Fig. 2:**
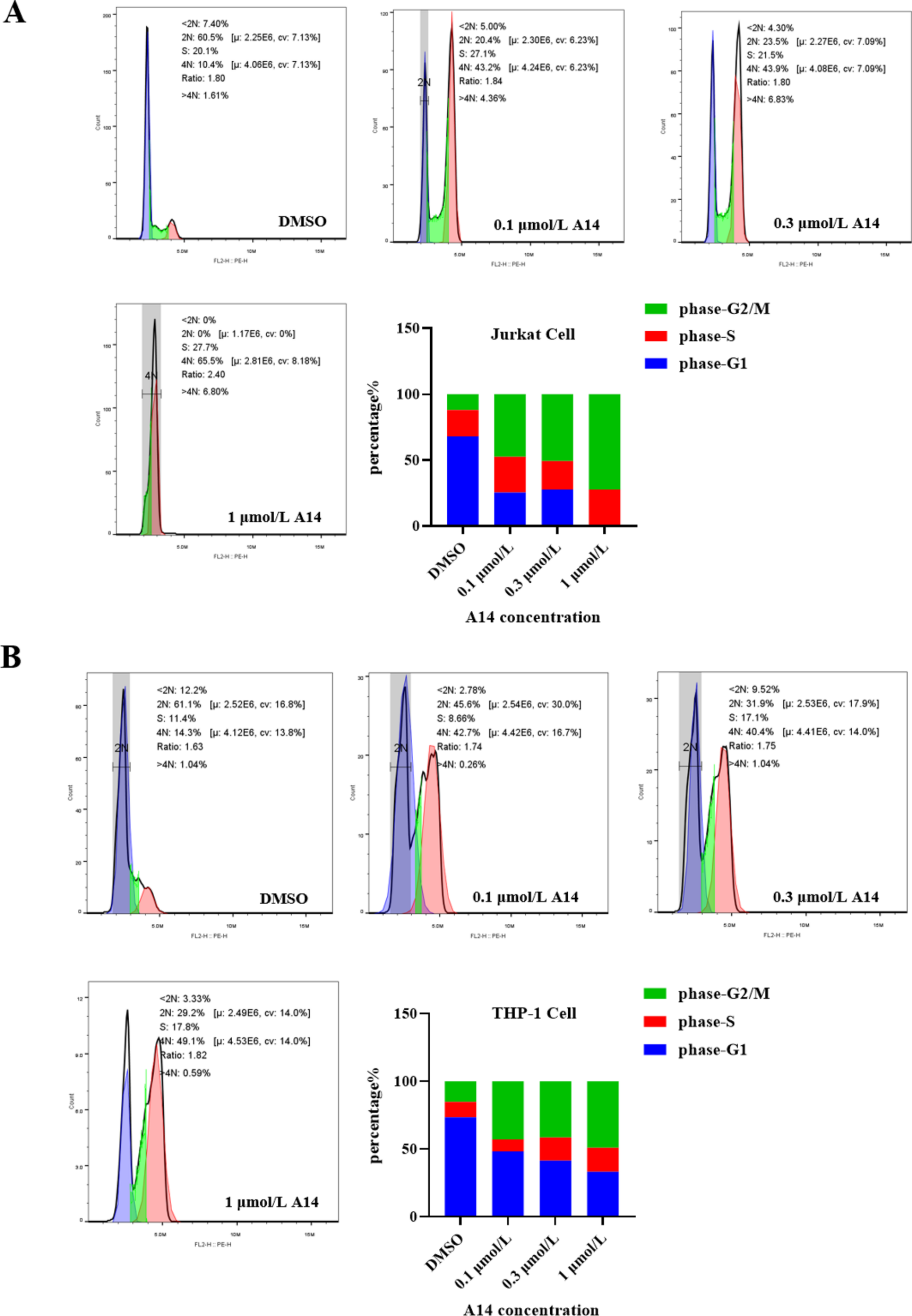
A14 induces cell cycle arrest in leukemia cells. **A** Jurkat cells were treated with different concentrations (0.1, 0.3 and 1 µmol/L) of A14 for 24 h and then analyzed by flow cytometry. **B** THP-1 cells were treated with different concentrations (0.1, 0.3 and 1 µmol/L) of A14 for 24 h and then analyzed by flow cytometry

The cell cycle is tightly associated with the cell proliferation. In order to explore the potential of A14 resistance to cell cycles, the cell cycle was validated with flow cytometers. As shown in Fig. 2, the cell cycles of both Jurkat and THP-1 cells were increasingly arrested in G2/M phase after A14 treatment compared with the control group (Fig. 2A) and THP-1 cells (Fig. 2B), indicating that leukemia cell cycles were blocked in the G2/M period after A14 challenge.

**Fig. 3:**
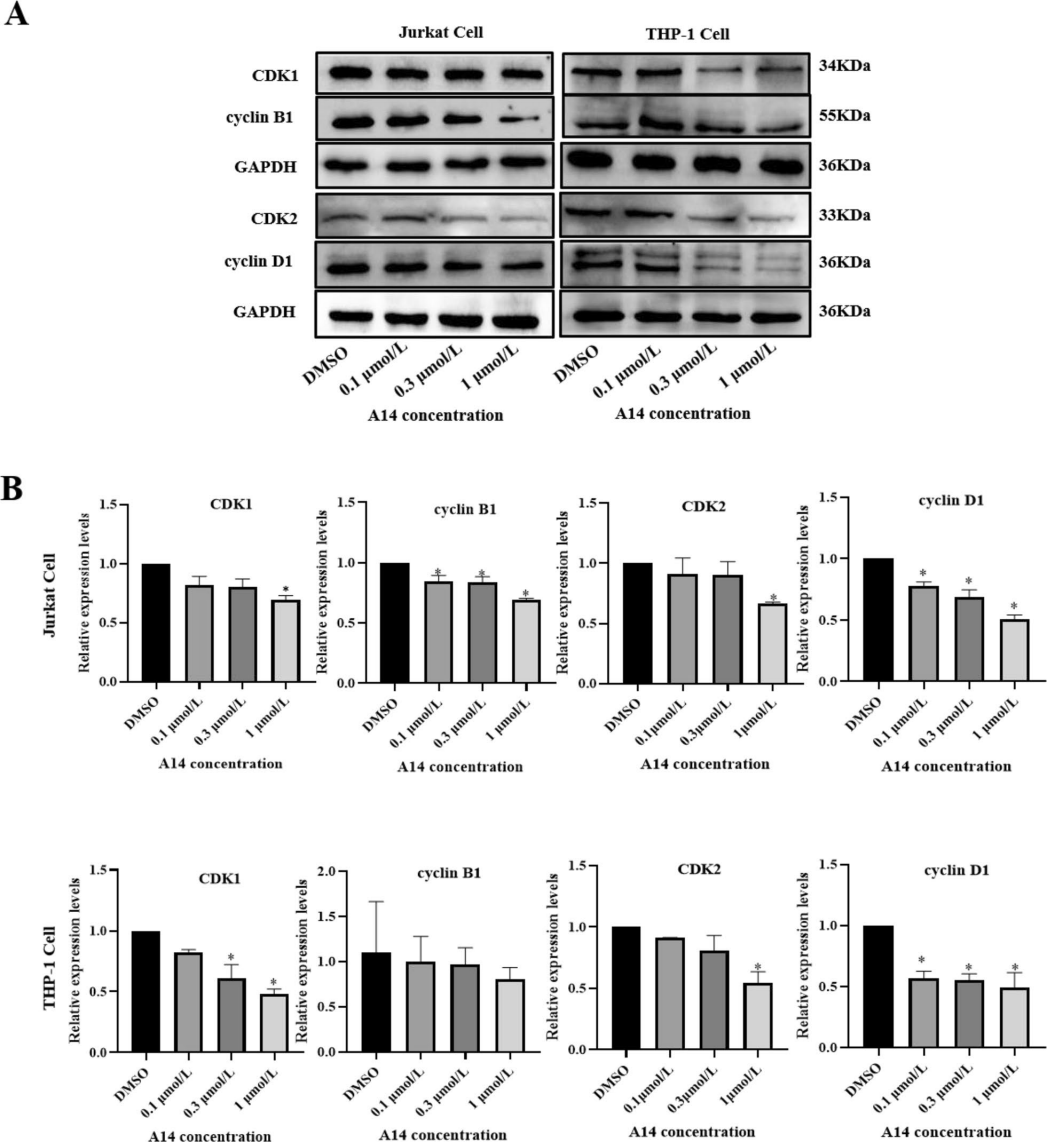
A14 ameliorates cell cycle proteins expression. **A** CDK1, cyclin B1, CDK2 and cyclin D1 protein expression were measured by western blotting. **B** The optical density of protein bands were calculated to GAPDH with setting the values of DMSO group as one. n = 3–4, values represent the means ± SD. *p < 0.05 *vs.* DMSO group

Cyclins and cyclin-dependent kinases (CDKs) work together in regulating cell cycles. In order to explore the cell cycle arrest caused by A14, we measured the protein expression of cyclins and CDKs by western blot. Results demonstrated that A14 treatment down-regulated the cyclin B1 and CDK1 expression in Jurkat cells and THP-1 cells. In addition, A14 challenge also suppressed cyclin D1 expression with inhibition of CDK2 expression (Fig. 3A and B).

### A14 treatment induces cell apoptosis

**Fig. 4:**
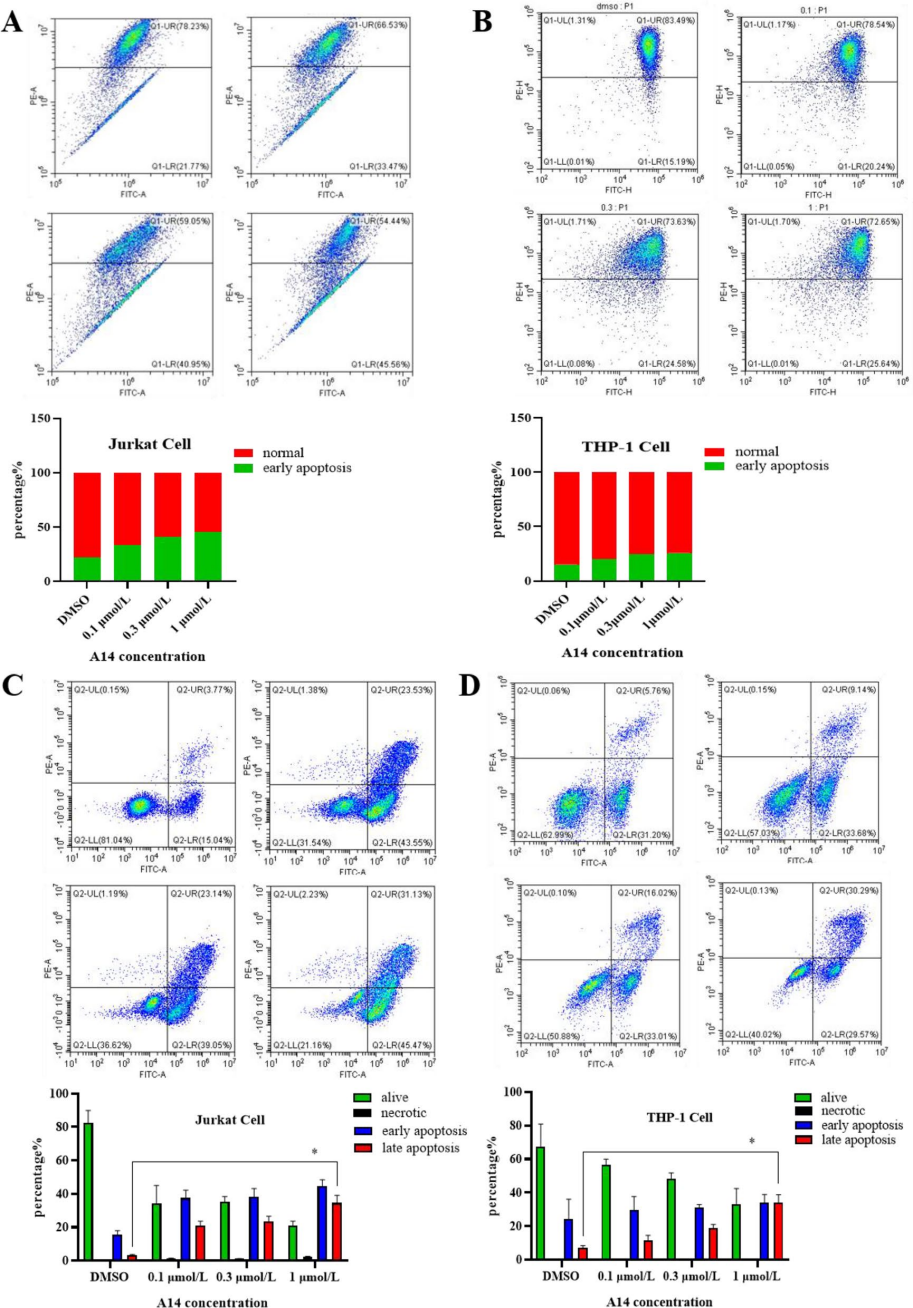
A14 induced cell apoptosis in leukemia cells. A14 induced the mitochondrial membrane potential changes in Jurkat cells (**A**) and THP-1 cells (**B**) were detected. Cell apoptosis was measured using flow cytometry in Jurkat cells (**C**) and THP-1 cells (**D**) n = 3–4, values represent the means ± SD. *p < 0.05 *vs.* DMSO group

It is well known that cell cycle arrestment usually leads to cells apoptosis, so the effect of A14 on cell apoptosis is a detectable phenomenon. The depolarization of mitochondrial membrane potential often indicates early apoptosis of cells, so we first detected the mitochondrial membrane potential changes. Results showed that the depolarization of mitochondrial membrane potential exhibited the elevation trend in Jurkat cells but there was no significant change in THP-1 cells when challenged with A14 (Fig. 4A and B). Consistently, flow cytometer results also demonstrated that the proportion of early apoptosis was enhanced in Jurkat cells, however, the proportion of late apoptosis in the two leukemia cells was increased after A14 treatment compared with the control group. These data suggest that A14 can cause apoptosis in leukemia cells (Fig. 4C and D).

**Fig. 5:**
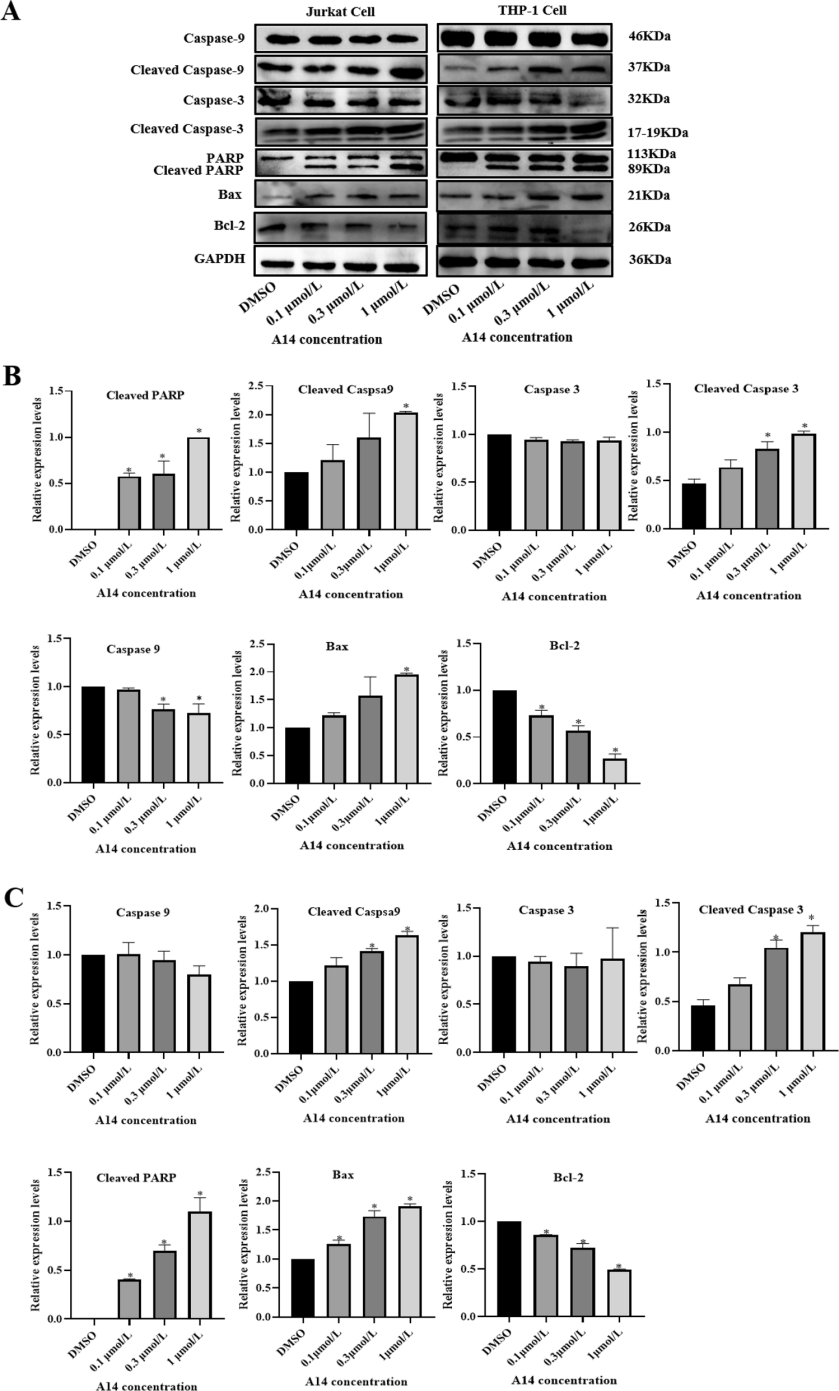
A14 ameliorates cell apoptosis related proteins expression. **A** Caspase-9, cleaved Caspase-9, Caspase-3, cleaved Caspase-3, PARP, cleaved PARP, Bax and Bcl2 protein expression were measured by western blotting. The optical density of protein bands in Jurkat cells (**B**) and THP-1 cells (**C**) were calculated to GAPDH with setting the values of DMSO group as one. n = 3–4, values represent the means ± SD. *p < 0.05, *vs.* DMSO group

Following our process with cell cycles, we also detected the protein expression associated with apoptosis signaling pathways using western blot. Results showed that A14 treatment could enhance cleaved caspase-3 and cleaved caspase-9 in Jurkat cells and THP-1 cells along with inducing the upregulation of caspase-3 downstream, thereby cutting substrate cleaved PARP compared to the control group in both leukemia cells, leading to PARP cutting (Fig. 5A–C). In addition, A14 also enhanced the pro-apoptotic protein Bax expression by inhibiting the expression of the anti-apoptotic protein Bcl-2 (Fig. 5A–C). These results indicate that A14 contributes to late apoptosis in leukemia cells and leads to cancer cell apoptosis at least partly through the cytochrome C signaling pathway.

### A14 ameliorates the survival rate in cell derived xenograft (CDX) model mice

**Fig. 6:**
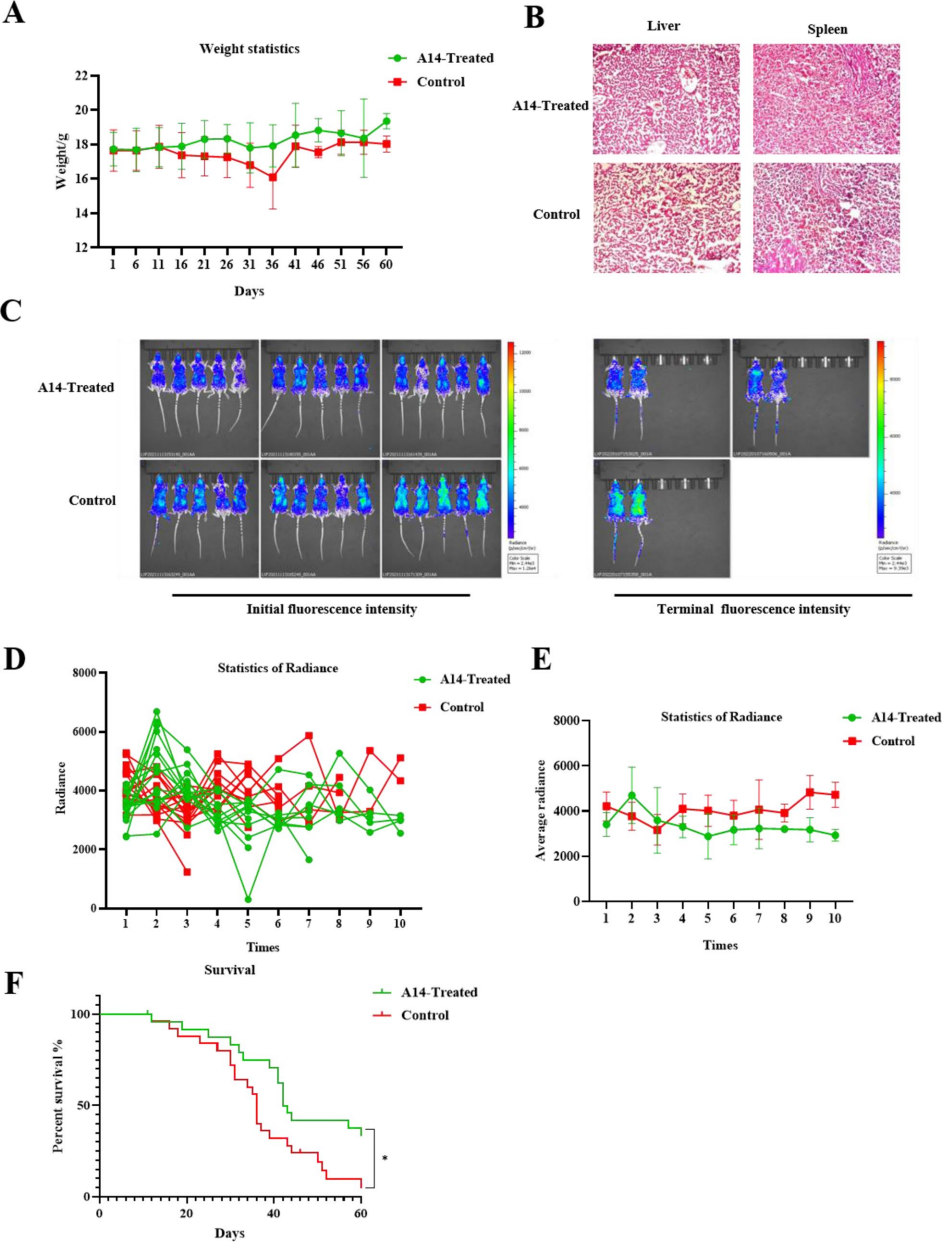
A14 protect against Jurkat cell derived xenograft model mice. **A** The body weight were recorded and calculated. **B** Representative hematoxylin and eosin (H&E) staining (100× magnification) of liver and spleen (n = 3–4). **C** The bioluminescence of mice was captured by IVIS Spectrum. **D** The fluorescence change of every mouse. **E** The average fluorescence change in control and A14-treated groups. **F** The survival rate in control and A14-treated groups. Values represent the means ± SD. *p < 0.05

We monitored and recorded the body weight of mice daily. At the end of the experiment, a line graph of the weight changes was drawn. As showed in (Fig. 6A), the overall weight of mice in the control group showed a downward trend, accompanied with death. Surprisingly, there were no significant changes of histopathological alteration in liver and spleen between the A14 treatment group and control group (Fig. 6B). To determine the density changes of Jurkat cells in mice, we detected the bioluminescence of mice using the IVIS Spectrum. The results exhibited that the fluorescence increase in the A14 treatment group was generally smaller than that in the control group compared with the initial fluorescence intensity of corresponding mice, suggesting that A14 could inhibit the proliferation of leukemia cells in CDX mouse model (Fig. 6C–E). We monitored and recorded the survival of each group of mice and plotted the survival curve. The results showed that A14 administration significantly extended the survival rate of mice compared to the control group. On the 52nd day all mice in the control group died, while 50% of the mice in the A14 treatment group survived (Fig. 6F), indicating that A14 may act as a promoting agent to protect against T-cell acute lymphoblastic leukemia.

## Discussion

The malignant transformation of T-cell precursors is major contributing factor to the etiology of T-ALL. Chemotherapy regimens with or without cranial radiation therapy are usually used for the treatment of patients with T-ALL in clinic [19]. In the present study, we investigated the effect of semisynthetic aurone A14 on T-ALL. Results showed that A14 suppressed leukemia cell proliferation by inducing G2 phase arrest and cell apoptosis. In addition, A14 also exhibited the capability to improve the survival rate of mice in the xenograft tumor model.

As is well known, vinca alkaloid vincristine is usually used as the standard component in the treatment of acute lymphoblastic leukemia, which mainly targets the β-tubulin subunit of αβ-tubulin heterodimers [20]. Drugs that target the colchicine binding site on tubulin provide a potential new approach in the treatment of T-ALL. Colchicine binds with tubulin and suppresses the microtubule assembly. Though colchicine itself is not used as an agent to protect against cancer, several tubulin inhibitors are investigated in preclinical or clinical trials for the interaction with the colchicine binding site [21]. Previous studies demonstrated that semisynthetic aurones inhibit tubulin polymerization at the colchicine-binding site [18], indicating the possible mechanism of action of A14 on T-ALL. Indeed, we found A14 significantly inhibited proliferation.

It has been demonstrated that tubulin-destabilizing agents induce the cell cycle arrest in the G2/M phase, which is associated with the cytoskeleton disruption and microtubule depolymerization [22]. G2/M phase arrest suppresses cancer cell proliferation by blocking the repair of damaged DNA, so the G2/M checkpoint is a potential therapeutic target [23]. We detected the effects of A14 on cell cycle distribution in Jurkat and THP-1 cell lines. Similar to other tubulin targeting compounds, A14 also blocked the G2/M phase, indicating that A14 may be a promising drug. It has been well known that different classes of cyclins and CDKs are tightly associated with cell cycle progression [24]. Cyclins are synthesized and broken down during the cell cycle, which is regulated by the kinase activity. Usually the interphase CDKs (CDK2, CDK4 and CDK6) and a mitotic CDK1 can interact with four kinds cyclins (cyclin A, cyclin B, cyclin D, cyclin E) to format the CDK–cyclin complexes. Changes in the CDK–cyclin complexes result in the continued proliferation of cancer cells [25]. Cell cycle progression is monitored by the checkpoints during chromosome segregation and DNA synthesis. The checkpoints regulate the CDK activity and induce cell cycle arrest. Complex CDK with cyclin D or cyclin E is responsible for the G1 phase during cell cycle. CDK2 and cyclin A are associated with the S-phase. Additionally, the complexes of CDK1 with cyclin A or cyclin B are required for the G2-phase and M-phase respectively. It has been demonstrated that the CDK1/ cyclin B1 complex is an important requirement for cell mitosis [26]. In our study, we measured the CDK1 and CDK2 protein expression along with the cyclin B and cyclin D alteration. Results demonstrated that A14 may inhibit cell proliferation through adjusting the cell cycle distribution.

Apoptosis is pivotal for physiological cell death in body. The signaling pathway of apoptosis is mainly divided into two paths. One is death receptor ligation signaling and the other is cytochrome c signaling. Briefly, the death receptor ligation signaling usually recruits the caspase-8 precursor to a death-inducing complex, leading to the activation of caspase-8 [27]. However, the cytochrome c signaling is usually stimulated by challenges such as radiation, drugs and so on in mitochondria. Cytochrome c is released from the mitochondria and binds to Apaf1 and ATP, leading to the activation of caspase-9 [28]. Previous studies have reported that the accumulation of α -tubulin acetylation activated the caspases involved in apoptosis progression [29]. In the present study, we found A14 induces cell apoptosis and relies, at least partly, on cytochrome c signaling.

## Conclusions

In conclusion, our study revealed that semisynthetic aurone A14 suppressed leukemia cell proliferation in vitro, in addition, A14 administration also extended the survival rate in cell derived xenograft mice model. Our results suggest that A14 is a promising agent for the treatment of T-ALL, and requires further investigation in patient derived xenograft models and T-ALL patients.

## Data Availability

All the data used to support the findings of this study are available from the corresponding author upon reasonable request.

## Abbreviations

ALL: Acute lymphoblastic leukemia
ANOVA: Analysis of variance
CCCP: Carbonyl cyanide 3-chlorophenylhy drazone
CDKs: Cyclin-dependent kinases
CDX: Cell derived xenograft
DMSO: Dimethyl sulfoxide
DMF: n,n-Dimethylformamide
DMEM: Dulbecco’s modified eagle’s medium
FBS: Fetal bovine serum
HRP: Horseradish peroxidase
PBS: Phosphate buffered saline
PVDF: Polyvinylidene fluoride
PI: Propidium iodide
RPMI 1640: Roswell Park Memorial Institute 1640
SDS-PAGE: Sodium dodecyl sulphate–polyacrylamide
SD: Standard deviation
